# Effect of supplemental oxygen on native blood and myocardial MOLLI T1 relaxation times

**DOI:** 10.1186/1532-429X-17-S1-W32

**Published:** 2015-02-03

**Authors:** James Goldfarb, Kathleen Gliganic, Nathaniel Reichek

**Affiliations:** 1 Research and Education, St Francis Hospital, Roslyn, NY, USA

## Background

Magnetic resonance (MR) T1 relaxation time measurements are increasingly used for myocardial tissue characterization. Significant differences in native and gadolinium-enhanced measurements have been associated with ischemic and non-ischemic cardiomyopathies, myocardial fat deposition, fibrosis and edema as well as regional and global ventricular functional parameters.

Supplemental oxygen is often given to cardiac MR patients for improved breatholding. High flow supplemental oxygen with a non-rebreather mask is reported to reduce both myocardial and blood T1 relaxation times and has been studied with HASTE and FLASH T1 relaxation measurements for the optimization of MR ventilation scanning. The primary mechanism is dissolved oxygen acting as a paramagetic contrast agent. Conversely other reports show an increase of blood T1 times with increasing oxygen saturation. We studied the effect of supplemental oxygen on myocardial and blood T1 relaxation times using a well-documented T1 MOdified Look-Locker Imaging (MOLLI) protocol.

## Methods

Twelve healthy subject without respiratory or cardiac disease (age: 47.4±5.3 years; 6 male) were studied at 1.5T using MOLLI T1 mapping (TE/TE= 2.8/1.2 ms; 3-(3)-5; 2 inversions, 3 heartbeat rest period; TI start=120 ms; TI increment=120ms; 3 parameter curve fitting). Images were acquired in the four chamber view. Five measurements spaced by 10 minutes were performed with supplemental oxygen supplied by nasal cannula and a non-reberather mask alternating with room air (M1: Room air, M2: nasal oxygen (2 l/m), M3: Room air, M4: non-rebreather mask (15 l/m), M5: room air). Regions-of-interest were drawn for T1 measurements in the boodpool of each ventricle and atria as well as septal myocardium. The effects of supplemental oxygen were investigated statistically using a mixed model analysis of variance.

## Results

**Figure 1:**
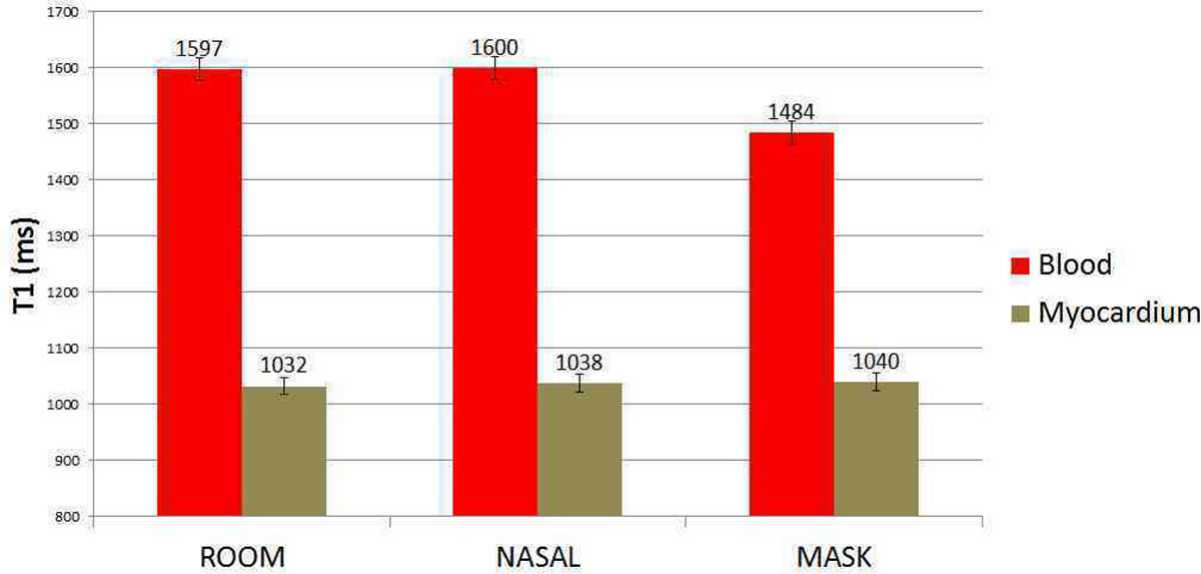
A significant difference in blood T1 was seen using a non-rebreather mask. A T1 change was not detected with nasal cannula or in the myocardium.

**Figure 2:**
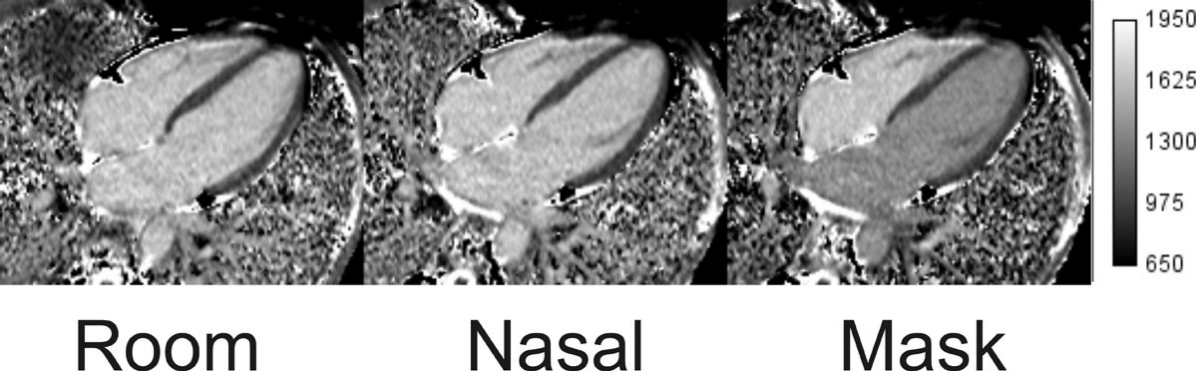
Native T1 maps with the subject breathing room air (ROOM), 2 l/m flow with nasal canula (NASAL) and 15 l/m with a non-rebreather mask (MASK). Reduced blood T1 with the non-rebreather mask can be visualised in the left side of the heart.

Study results are displayed in Figure 1. In contrast to previously published reports, there was no difference between myocardial T1 relaxation times; p=.28 with supplemental oxygen. There was a significant difference between blood T1 relaxation times; p<0.001. Left ventricular and atrial T1 bloodpool relaxation times were significantly reduced using the non-rebreather mask and could be easily visualized in source T1 maps (Figure 2). There was no detected change in right atrial or ventricular bloodpool T1 times with supplemental oxygen, p>0.05.

## Conclusions

Use of supplemental oxygen can change measured MR T1 relaxation values. As measured using a well-documented MOLLI T1 protocol, there are significant changes in left ventricular and atrial T1 relaxation times with supplemental oxygen supplied by a non-rebreather mask. We did not detect a change in oxygen supplied by nasal cannula. Additionally, myocardial, right atrial and ventricular bloodpool T1 relaxation times did not change with supplemental oxygen. If supplemental oxygen is used, one can measure blood relaxation times from the right side of the heart as they are unaffected.

